# The relationship of trait-like compassion with epigenetic aging: The population-based prospective Young Finns Study

**DOI:** 10.3389/fpsyt.2023.1018797

**Published:** 2023-04-18

**Authors:** Henrik Dobewall, Liisa Keltikangas-Järvinen, Saara Marttila, Pashupati P. Mishra, Aino Saarinen, C. Robert Cloninger, Igor Zwir, Mika Kähönen, Mikko Hurme, Olli Raitakari, Terho Lehtimäki, Mirka Hintsanen

**Affiliations:** ^1^Faculty of Education, VISE Research Unit, Faculty of Education and Psychology, University of Oulu, Oulu, Finland; ^2^Finnish Institute for Health and Welfare, Helsinki, Finland; ^3^Department of Psychology and Logopedics, Faculty of Medicine, University of Helsinki, Helsinki, Finland; ^4^Molecular Epidemiology, Faculty of Medicine and Health Technology, Tampere University, Tampere, Finland; ^5^Gerontology Research Center, Tampere University, Tampere, Finland; ^6^Fimlab Laboratories and Finnish Cardiovascular Research Center - Tampere, Faculty of Medicine and Health Technology, Tampere University, Tampere, Finland; ^7^Department of Psychiatry, Washington University School of Medicine, St. Louis, MO, United States; ^8^Department of Computer Science, University of Granada, Granada, Spain; ^9^Department of Clinical Physiology, Tampere University Hospital and Faculty of Medicine and Health Technology, Tampere University, Tampere, Finland; ^10^Department of Microbiology and Immunology, Faculty of Medicine and Health Technology, Tampere University, Tampere, Finland; ^11^Research Centre of Applied and Preventive Cardiovascular Medicine, University of Turku, Turku, Finland; ^12^Department of Clinical Physiology and Nuclear Medicine, Turku University Hospital, Turku, Finland; ^13^Centre for Population Health Research, University of Turku and Turku University Hospital, Turku, Finland

**Keywords:** compassion, temperament and character inventory, DNA methylation, epigenetic aging, DNAmPhenoAge, prosocial personality, longevity

## Abstract

**Introduction:**

Helping others within and beyond the family has been related to living a healthy and long life. Compassion is a prosocial personality trait characterized by concern for another person who is suffering and the motivation to help. The current study examines whether epigenetic aging is a potential biological mechanism that explains the link between prosociality and longevity.

**Methods:**

We used data from the Young Finns Study that follows six birth-cohorts from age 3–18 to 19–49. Trait-like compassion for others was measured with the Temperament and Character Inventory in the years 1997 and 2001. Epigenetic age acceleration and telomere length were measured with five DNA methylation (DNAm) indicators (DNAmAgeHorvath, IEAA_Hannum, EEAA_Hannum, DNAmPhenoAge, and DNAmTL) based on blood drawn in 2011. We controlled for sex, socioeconomic status in childhood and adulthood, and body-mass index.

**Results and discussion:**

An association between higher compassion in 1997 and a less accelerated DNAmPhenoAge, which builds on previous work on phenotypic aging, approached statistical significance in a sex-adjusted model (*n* = 1,030; *b* = −0.34; *p* = 0.050). Compassion in 1997 predicted less accelerated epigenetic aging over and above the control variables (*n* = 843; *b* = −0.47; *p* = 0.016). There was no relationship between compassion in 2001 (*n* = 1108/910) and any of the other four studied epigenetic aging indicators. High compassion for others might indeed influence whether an individual’s biological age is lower than their chronological age. The conducted robustness checks partially support this conclusion, yet cannot rule out that there might be a broader prosocial trait behind the findings. The observed associations are interesting but should be interpreted as weak requiring replication.

## 1. Introduction

### 1.1. Compassion as a personality trait and its association with longevity

There is accumulating evidence that prosocial behavior, such as helping others, providing social support, and caregiving within and beyond the family, is associated with better health, wellbeing, and longevity (1–4). It has been shown that the motive behind prosocial behavior is important. Only individuals who help for other-oriented, non-egoistic reasons have a mortality advantage (5). Consequently, individuals’ relatively stable personality traits have been recognized as predictors of longevity (6–9). Knowledge about the underlying biological mechanisms is growing (4, 10–13), although less is known about whether individual differences in prosociality predict the pace of epigenetic aging.

The current study focuses on trait-like compassion, which can be characterized by concern for another person who is suffering and the motivation to help (14, 15). Compassion for others has developed from a caring motivation that, over the course of evolution, was extended beyond the family (14, 16) as it has health benefits and survival value (16). Garcia et al. (17) describe compassionate individuals as forgiving, charitable, and benevolent. They try to be constructive in a relationship and when dealing with interpersonal conflicts, rather than seeking revenge and reacting in a hostile manner (17, 18). An individual’s authentic desire to alleviate undeserved human suffering can lead to prosocial behavior (19, 20) if accompanied by commitment and the competencies needed in a given situation (16, 21). Compassion is a subscale belonging to the broader character trait Cooperativeness in Cloninger’s biopsychological model of personality, which measures how well an individual gets along with others (17). Cooperativeness has clear links to the five-factor model of personality which has, like compassion, well-studied influences on health and well-being (8, 12, 22, 23). Moreover, more agreeable individuals were found to have lower mortality risk (6, 7, 9), whereas a more hostile and vulnerable personality is inversely associated with longevity (7, 24). Epigenetic aging is a potential biological mechanism that might explain the link between prosociality and longevity, investigated in this work by the example of compassion for others.

### 1.2. Epigenetic aging

The biological age of an individual can be different than the date on his or her birth certificate (25, 26). The variance in biological age left unexplained by chronological age is thus a potential mechanism for the hypothesized effect of compassion on longevity. Biomarkers of aging have been defined as individual lifespan differences in time to the onset of disease, decline in functional capacity, and death (27). One of the first indicators of epigenetic aging was found in telomere biology (28). Telomeres are the protective ends of chromosomes that shorten at each cell division. Telomere length is consequently strongly associated with chronological age (29). More recently, epigenetic clocks were developed as biomarkers of aging by building on the finding that DNA methylation (DNAm) patterns change across the genome as an individual grows older (30, 31). If the biological age is higher than the chronological age, this refers to an accelerated epigenetic age (26, 31, 32). DNAm age acceleration has been found to have detrimental effects on aging-related health conditions, cognitive and physical functioning, and cause-specific and all-cause mortality (25–27, 33, 34). It should however be noted that telomere biology and methylation-based age acceleration share only little variance (26), and thus appear to be linked through independent pathways to aging-related health conditions and mortality risk (34).

Since DNAm biomarkers capture epigenetic changes that are probably reversible (25), a better understanding of the causes and consequences of epigenetic aging might make it possible to prevent aging-related chronic diseases and thus to prolong the human lifespan (35, 36). However, relatively little is known about the association between motivational prosocial personality traits and epigenetic aging. It is therefore important to examine whether compassion for others triggers biological mechanisms that lead to longevity.

Although there is no previous research on trait-like compassion for others and epigenetic aging, we can draw on previous evidence for practicing compassion, even though self-compassion and other-compassion are distinct constructs (22, 37). With regard to loving-kindness training, which cultivates warm and friendly feelings toward oneself, close others, and more distant persons, it was found that it had a buffering effect on telomere shortening which was stronger than for mindfulness training, which is less related to compassion (38). Meditators, practicing either mindfulness or compassion-related meditation, were also found to have slower running epigenetic clocks in old age (35). Further, the effect of meditation including compassion practices on telomerase activity was found to be partially transmitted through changes in personality traits (39).

### 1.3. The current study

The current study examines whether the motivational personality trait compassion for others predicts DNAm-based epigenetic age acceleration. Previous studies in the compassion domain relied mostly on cross-sectional data or relatively short and not very intense interventions, whereas we assessed compassion as a trait longitudinally with a personality measure covering developmental changes from the age of 20 to 39 years (40).

Over the past three decades, several types of biological age indicators have been proposed, and DNA methylation-based epigenetic clocks outperform other potential biomarkers of aging in terms of validity and predictive power (25, 26). We include five indicators of biological aging (DNAmAgeHorvath, IEAA_Hannum, EEAA_Hannum, DNAmPhenoAge, and DNAmTL) to reflect the different aspects of the human aging process (26, 27, 31, 33, 41). They were assessed in a large and representative sample, whereas previous studies on practicing compassion (not compassion as a trait) included only a single measure of epigenetic age acceleration and had the limitation of small sample sizes (e.g., Ref. (35)). We also attempted to investigate whether the association found between practicing compassion and telomere length holds for compassion as a trait (38, 39).

The multi-generational, genetically informed nature of our data supports testing whether compassion predicts a less accelerated epigenetic age over and above childhood, and adulthood risk factors, such as socioeconomic status and body-mass index.

## 2. Methods

### 2.1. Participants

The Young Finns Study (YFS) is an ongoing prospective multidisciplinary study that follows six birth cohorts from childhood through adulthood (42). Participants living in an area with a university hospital were selected from the Finnish population registry. The original sample consisted of 3,596 individuals. The participants were 3, 6, 9, 12, 15, and 18 years old at baseline (1980, T0) and they answered the compassion scale in 1997 (T1, youngest participant 20 years old) and 2001 (T2, oldest participant 39 years old). Blood samples were drawn from the participants in the year 2011 (ages 19–49 years).

The YFS was approved by all participating universities’ ethics committees at the beginning of the study in 1980, and the follow-ups were approved by the ethics committee of the University of Turku [vernacular institution name: Varsinais-Suomen sairaanhoitopiirin kuntayhtymä, Eettinen toimikunta, Meeting Number 9/2010; study name, “Lasten sepelvaltimotaudin riskitekijät projekti (Laseri) 30-vuotis seurantatutkimus, 25.8.2010”]. The YFS was conducted according to the guidelines of the Helsinki declaration. Written informed consent to participate in the YFS was obtained. Summary statistics for all the main study variables can be found in Table 1.

**Table 1:** Summary statistics of main study variables.

Variable	*n*	Mean	*SD*	Min	Max
Epigenetic age acceleration Horvath	1273	−0.04	4.21	−22.69	19.49
Intrinsic epigenetic age acceleration Hannum	1273	−0.02	4.09	−19.56	18.93
Extrinsic epigenetic age acceleration Hannum	1273	−0.09	5.15	−18.71	17.28
Phenotypic epigenetic age acceleration	1273	−0.04	5.33	−17.47	20.11
DNAm indicator of telomere length	1273	0.01	0.18	−0.62	0.60
Compassion 1997	1,030	0.04	0.97	−3.26	2.16
Compassion 2001	1,108	0.03	0.97	−4.10	2.02
Gender (male)	1273	0.42	0.49	0.00	1.00
Socioeconomic status adulthood	1130	−0.01	0.97	−2.55	1.91
Socioeconomic status childhood	1197	0.03	1.01	−1.51	2.28
Body-mass index	1218	0.01	1.00	−1.95	6.11

### 2.2. Compassion measure

Trait-like compassion for others was measured with the Temperament and Character Inventory (TCI), developed and validated by Cloninger and colleagues (18, 43). The compassion (versus revengefulness) scale has 10 items (e.g., “I like to imagine my enemies suffering” [reverse scored]; “It gives me pleasure to help others, even if they have treated me badly” [positively scored]; “It gives me pleasure to see my enemies suffer” [reverse scored]; and “I hate to see anyone suffer” [positively scored]). Compassion for others belongs to the broader cooperativeness scale consisting of 42 items in total, the other subscales being helpfulness (versus unhelpfulness), empathy (versus social disinterest), social acceptance (versus social intolerance), and pure-hearted conscience (versus self-serving advantage) (17). We used the revised TCI answered on a Likert-scale with five response options (44). We rescored the reverse-scored items before the mean for each measurement occasion was calculated. We excluded participants if they had responded to less than 50% of the compassion items. The compassion scale has a high internal consistency (Cronbach’s αT1-T2 ≥ 0.86) and is relatively stable over time (rT1- > T2 = 0.71; *p* < 0.001). It has a good model fit when accounting for the common variance between the reverse-scored items (10, 23). Due to the age-dependency of compassion (40), we standardized the mean scores within the six birth cohorts.

### 2.3. Epigenetic clocks

DNA methylation (DNAm) age was calculated successfully for 1,529 participants based on whole blood data measured by utilizing the Illumina Infinium platform from 2011 EPIC/850 K array. The sample size of the main analyses is smaller (*n* = 843–1,108) because not all participants filled in the psychological questionnaires. We report results for five DNAm age indicators that were estimated with an online calculator^1^. The underlying method and R function are described in Horvath (31). The produced indicators were normalized, as this improves the predictive accuracy and makes the data more comparable to the training data (32). The average correlation between the samples and the gold standard was good (*r* ~ 0.97). In 60.6% of the samples the predicted tissue was correct and sex was always correctly predicted. This information indicates a high precision of the DNAm age predictions.

These DNAm based biomarkers can be broadly categorized into (cell-)intrinsic and extrinsic measures of epigenetic aging (25, 26, 33).

#### 2.3.1. Intrinsic DNAm age measures

We included two intrinsic DNAm age indicators. Intrinsic indicators are largely unperturbed by and independent of cell-type composition accompanied by aging ((25); for some contrary evidence see (45)), and are thought to represent an epigenetic maintenance system (26, 31). The DNAmAge indicator of Horvath (31) is estimated based on 353 CpGs and can predict age across multiple tissues and cell types. The indicator has high accuracy already in childhood and adolescence (46). The difference between DNAmAgeHorvath and chronological age was associated with a higher all-cause mortality risk assessed 5 years later, even after accounting for sociodemographic background, childhood IQ, and other risk factors (47). The validity of DNAmAgeHorvath was further demonstrated by associations with physical and cognitive fitness (48) and cancer and cardiovascular mortality (49). Another epigenetic clock has been developed by Hannum et al. (30). This blood-based indicator of aging-related methylation levels uses 71 CpGs. Based on Hannum’s clock the intrinsic epigenetic age acceleration (IEAA_Hannum) indicator has been developed, which is by design not confounded by differences in blood cell counts (33). IEAA_Hannum has also shown many robust associations with diverse aging-related conditions (25, 33). As intrinsic DNAm age measures have been around for longer than their extrinsic complements, they are probably the most researched epigenetic clocks in the literature.

#### 2.3.2. Extrinsic DNAm age measures

We also included two extrinsic DNAm indicators of epigenetic aging. Extrinsic DNAm age indicators are more dependent on aging-related changes in blood cell counts and appear to be associated with immune senescence (27, 33, 50).

The extrinsic epigenetic age acceleration (EEAA_Hannum) is the second enhanced indicator based on Hannum’s clock (33). EEAA_Hannum is a strong predictor of time to death across ethnic groups (33), assessing the aging-related functional decline of the immune system. A recently identified epigenetic biomarker is the phenotypic age estimator (DNAmPhenoAge), which was proposed by Levine et al. (27) building on previous work on phenotypic aging. It taps into physiological dysregulation in aging individuals and has been developed by predicting a composite indicator of nine morbidity-and mortality-risk factors (e.g., albumin levels and white blood cell count) and chronological age, and was estimated based on 513 CpGs. DNAmPhenoAge was classified by Horvath and Raj (25) as belonging to the group of extrinsic DNAm age indicators. DNAmPhenoAge has been found in recent reviews and meta-analyses to predict future aging-related diseases and mortality better than the above epigenetic clocks (25, 27).

There is some evidence that extrinsic measures are under weaker genetic control and are thus more influenced by the environment than intrinsic measures (47, 51). Finally, extrinsic measures of epigenetic aging were found to be more strongly associated, compared to intrinsic measures, with demographic variables and external factors that are under behavioral control (25).

For all four epigenetic age acceleration indicators, the residuals were used that resulted from regressing the calculated DNAm age on chronological age.

#### 2.3.3. DNAm age measure of telomere length

We included a DNA methylation-based indicator of telomere length (DNAmTL) estimated with 140 CpGs (41). DNAmTL correlates with measured leukocyte telomere length (r ~ −0.35) and outperforms the latter in predicting time to death and coronary heart disease or heart failure (41). It is thought to provide additional insights into mechanisms linking exposure to environmental factors, cell replication, and aging-related health conditions and mortality risk. We used the age-adjusted version of DNAmTL.

### 2.4. Control variables

Following previous studies, we included standard control variables (see Ref. (52)). We adjusted for sex, socioeconomic status in childhood (SESC in 1980) and adulthood (SESA in 2007) (i.e., composite scores of high educational level, upper white-collar occupation, currently having a job, and highest income quartile) (53), and body-mass index (BMI) (54). The control variables were standardized within the six birth cohorts before filling the missing values with measurements of the same variables from a later YFS wave (SESC in 1983, SESA 2012, BMI 2012) in order to increase the sample size in the adjusted analyses.

### 2.5. Analytical strategy

All analyses were run with Stata version 15.1.

First, we tested the degree to which selective attrition might have affected our results by means of chi-square tests and independent samples *t*-test. Included participants donated blood samples and responded to the compassion scale at least once.

Then, we computed the correlations between the main study variables.

The main analyses of the effect of compassion on DNAm age acceleration and DNAmTL were conducted with multivariate multiple regression. The analyses were conducted separately for compassion for others in 1997 and 2001. In the first step, we controlled the association only for sex (N1997/2001 = 1,030 / 1,108). In the second step, we included childhood and adulthood SES as well as BMI (N1997/2001 = 843 / 910). For each methylation-based indicator of biological aging, we ran separate linear regression analyses.

To assess the robustness of our findings, we further tested whether broader prosocial tendencies in cooperativeness or one of the other subscales are driving them, whether they are dependent on the balanced wording of the compassion scale, whether they hold when controlling baseline levels of the outcome, and whether they are robust to variation in sample size.

## 3. Results

### 3.1. Preliminary analyses

Attrition analysis indicated that excluded participants had lower DNAmTL (mean = −0.03 vs. 0.01; *p* = 0.004) and were more likely to be male (53% vs. 42%, *p* < 0.001) than included participants who were required to respond to the TCI at least once and donated a blood sample. Other study variables were not affected by selective attrition.

The epigenetic clocks were moderately to strongly correlated with one another but not with compassion in the unadjusted analyses (Table 2). Men had higher methylation-based biological age measured with DNAmAgeHorvath, IEAA_Hannum, and EEAA_Hannum than women. Their DNAmPhenoAge acceleration, however, was lower and they had shorter DNAmTL. Of the five indicators, only younger DNAmPhenoAge was associated with higher socioeconomic status both in childhood and adulthood, whereas all five DNAm age indicators were associated with the body-mass index.

**Table 2:** Pearson correlations between the main study variables.

	1	2	3	4	5	6	7	8	9	10	11
1) Epigenetic age acceleration Horvath	1										
2) Intrinsic epigenetic age acceleration Hannum	**0.97**	1									
3) Extrinsic epigenetic age acceleration Hannum	**0.31**	**0.28**	1								
4) Phenotypic epigenetic age acceleration	**0.32**	**0.32**	**0.47**	1							
5) DNAm indicator of telomere length	**−0.14**	**−0.06**	**−0.36**	**−0.32**	1						
6) Compassion 1997	−0.05	−0.05	−0.03	−0.05	0.01	1					
7) Compassion 2001	−0.02	−0.02	0.02	0.01	0.01	**0.68**	1				
8) Sex (male)	**0.17**	**0.13**	**0.19**	**−0.07**	**−0.20**	**−0.14**	**−0.14**	1			
9) Socioeconomic status adulthood	−0.01	−0.01	−0.04	**−0.10**	0.05	**0.08**	**0.08**	**0.04**	1		
10) Socioeconomic status childhood	−0.01	−0.01	−0.03	**−0.06**	0.05	0.03	**0.04**	0.00	**0.27**	1	
11) Body-mass index	**0.09**	**0.10**	**0.09**	**0.12**	**−0.06**	**−0.08**	−0.03	**0.14**	**−0.07**	**−0.09**	1

Associations marked in bold are statistically significant at *p* < 0.05 level.

### 3.2. Main analyses

The main results are presented in Table 3. The prediction of a less accelerated DNAmPhenoAge by higher compassion for others in 1997 approached statistical significance when controlling for sex alone (*p* = 0.050; Model 1; *n* = 1,030). Compassion in 1997 predicted epigenetic age acceleration even more strongly when further accounting for SESC, SESA, and BMI (*b* = −0.47; *p* = 0.016; Model 2, *n* = 843).

**Table 3:** Linear regressions of compassion in (a) 1997 and (b) 2001 Predicting five DNAm epigenetic aging indicators in 2011.

(a) Compassion in 1997	Model 1				Model 2			
	Beta	*p* value	*R* ^2^	*n*	Beta	*p* value	*R* ^2^	*n*
*Epigenetic age acceleration Horvath*			3%	1,030			4%	843
Compassion	−0.13	0.336			−0.10	0.529		
*Intrinsic epigenetic age acceleration Hannum*			2%				3%	
Compassion	−0.16	0.229			−0.14	0.352		
*Extrinsic epigenetic age acceleration Hannum*			2%				3%	
Compassion	−0.04	0.810			−0.05	0.787		
*Phenotypic epigenetic age acceleration*			2%				4%	
Compassion	−0.34	0.050			−0.47	0.016		
*DNA methylation-based indicator of telomere length*			2%				3%	
Compassion	0.00	0.784			0.00	0.851		
(b) Compassion in 2001
*Epigenetic age acceleration Horvath*			2%	1,108			4%	910
Compassion	−0.01	0.939			0.02	0.882		
*Intrinsic epigenetic age acceleration Hannum*			1%				3%	
Compassion	0.00	0.996			0.03	0.830		
*Extrinsic epigenetic age acceleration Hannum*			3%				4%	
Compassion	0.27	0.095			0.24	0.180		
*Phenotypic epigenetic age acceleration*			1%				5%	
Compassion	−0.03	0.850			−0.06	0.755		
*DNA methylation-based indicator of telomere length*			3%				4%	
Compassion	0.00	0.699			0.00	0.880		

Model 1 controls for sex and Model 2 includes controls for childhood socioeconomic status 1980, adulthood socioeconomic status 2007, and body-mass index 2007.

There were no associations between compassion in 1997 and the other four DNAm age indicators. Compassion in 2001 did not predict epigenetic aging (*p* > 0.09; *n* = 1,108 / 910).

Compassion and sex alone accounted for 0.9–2.9% of the variance in the part of epigenetic age left unexplained by chronological age. All the included predictors together explained 2.6–4.9% of the variance in the five DNAm age indicators.

### 3.3. Robustness checks

We found mixed empirical support for the interpretation that higher compassion predicts whether individuals have slower-running epigenetic clocks. We therefore conducted a series of robustness checks.

First, the reviewed literature makes it plausible that broader prosocial tendencies contribute at least partially to the observed findings. To test this possibility, we repeated the analyses for the full cooperativeness scale of the TCI. In 1997, this broader character trait capturing an individual’s prosociality predicted DNAmPhenoAge even more strongly than compassion (sex-adjusted, *b* = −0.48; *p* = 0.034 and fully-adjusted, *b* = −0.64; *p* = 0.012; Supplementary Table S1).

Second, we showed that it was compassion and not one of the other subscales that drove the reported findings. Associations for the other subscales were non-significant except for pure-hearted conscience in the sex-adjusted model, *b* = −0.38; *p* = 0.030, and empathy in the fully-adjusted model, *b* = −0.41; *p* = 0.044 (Supplementary Table S2).

Third, due to the balanced wording of our measure of compassion (versus revengefulness), it is possible that it reflects other concepts such as anger and hostility. We thus repeated the main analyses after excluding all negatively worded items belonging to the revengefulness pole (Supplementary Table S3). The balanced wording of the compassion scale did not affect the interpretation of the results considerably. A scale using only the positively worded items of the compassion pole showed the same pattern of association with DNAmPhenoAge as in the main analysis (sex-adjusted, *b* = −0.29; *p* = 0.092; fully adjusted, *b* = −0.44; *p* = 0.023).

Forth, to make it less likely that epigenetic age predicts compassion than vice versa and to make the presence of spurious effects less likely, we controlled for baseline levels in the outcome measured 25 years earlier in a smaller subsample (*n* = 66–81). We were able to confirm that DNAmPhenoAge was predicted by compassion in 1997 when accounting for epigenetic age in 1986 and sex (*b* = −1.50; *p* = 0.007), but when including all control variables, the effect only approached statistical significance (*p* = 0.052; Supplementary Table S4).

Fifth, we conducted a full case analysis to determine whether the reduction of sample size accounted for some of the findings. Now, the sex-adjusted marginally significant effect of compassion in 1997 on DNAmPhenoAge became statistically significant (*b* = −0.54; *p* = 0.006, *n* = 843). When imputing the missing values for all predictors with chained equations (MICE), however, the fully-adjusted effect became non-significant (*p* > 0.11; *n* = 1,030).

## 4. Discussion

The main finding of the current study was that the prediction of higher trait-like compassion of epigenetic age acceleration assessed with DNAmPhenoAge approached statistical significance in a gender-adjusted model. The phenotypic age estimator is a DNA methylation-based indicator that builds on extensive previous work on phenotypic aging. The prediction became stronger when controlling for socioeconomic status in childhood as well as adulthood, and BMI. To our best knowledge, this is however the first time that a potential biological aging mechanism has been found that links trait-like compassion for others to longevity. One explanation behind our finding is that practicing compassion helps to adapt to stress (55, 56) by directly improving emotion regulation skills and behavioral responses (56, 57). The better innate immune responses to stress of individuals high in compassion (56) might further be explained by the association between DNAm age acceleration and immune senescence (27, 50). An alternative explanation is that the emotion-regulation and calming aspects of trait-like compassion are some of the psychological mechanisms explaining how meditation training affects epigenetic aging (58). Compassion for others, for instance, reduces stress by increasing the ability to receive social support (59), and protects against vital exhaustion (a marker of susceptibility to stress), negative emotionality (a marker of chronic stress responses) (60), and elevated levels of cytokines (a marker of immune response) (12). Our main finding is also in line with the association observed between meditation training and telomere biology (38, 39). In summary, the current study examined the relationship between the motivational personality trait compassion and epigenetic aging, and found some first important and interesting evidence that the concern for another person who is suffering and the motivation to help might indeed lead to longevity.

We established the association with compassion when first measured, but were unable to replicate it with our later compassion measurement. Thus, results were dependent on the age of the participants (20–35 years vs. 24–39 years) when compassion was assessed. The role of compassion for others in epigenetic aging must thus be interpreted tentatively. It is well documented that trait-like compassion increases with age (40). One potential explanation for this is that there might not have been enough variance in compassion left in the second measurement to find statistically significant differences. Our study results require replication in an independent sample before more certain conclusions can be drawn.

We also tested whether the observed associations are specific for compassion or whether they apply to broader motivational, prosocial personality traits. A less accelerated epigenetic age was also predicted by Cloninger’s broader character trait cooperativeness, that captures an individual’s prosociality (17, 18). The association became even stronger. Thus, these further analyses showed that we cannot rule out that a broader prosociality trait accounted for at least part of the observed relationship between compassion and epigenetic aging.

We showed that the association was driven by the items capturing compassion and not revengefulness. The balanced wording of our measure of compassion did not account for the observed findings, given that personality concepts such as anger and hostility were previously found to be inversely associated with longevity (7, 24).

Our study also contributes to the accumulating evidence that prosocial behavior, such as helping others, providing social support, and caregiving within and beyond the family, is associated with better health, well-being, and longevity (1–4). It emphasizes that the motive behind prosocial behavior is important (5–7, 9).

Compassion as a personality trait did not have an effect on DNAmAgeHorvath, IEAA_Hannum, or EEAA_Hannum. Our null finding for most included DNAm-based indicators has implications for the development of anti-aging interventions. Both intrinsic and extrinsic epigenetic age acceleration estimators have previously been found to be associated with practicing compassion (35) and diverse lifestyle factors (61). On the basis of the review of previous literature (25, 51), it was expected that trait-like compassion would be more strongly related to (cell-) extrinsic DNAm indicators of epigenetic aging that appear more malleable, such as DNAmPhenoAge, than intrinsic indicators. There is some evidence that extrinsic measures are under weaker genetic control (47, 51) and show associations with external factors that are under behavioral control (25). DNAmPhenoAge is also special in the sense that it has been created to model biological age by building on a set of phenotypic morbidity-and mortality-risk factors (27). DNAmPhenoAge therefore captures the social gradient in health better than the other indicators, as shown by the associations with both childhood and adulthood socioeconomic status and BMI (e.g., (62)). This might in part explain why compassion was especially predictive when including the full set of social and health control variables. The other DNAm age indicators, by contrast, have been created with the aim of modeling chronological age, while assuming that the variation from chronological age represents biological age (i.e., DNAm age acceleration) (26, 31, 32). Because in the case of DNAmPhenoAge biological age is generated first and then methylation is used to model it, the logic behind this indicator is more robust and requires fewer assumptions than the other DNAm age indicators.

Previous research on practicing compassion reported inverse associations with telomere shortening (38, 39). To our best knowledge, this study pioneers the use of a DNA methylation-based indicator to assess telomere length and compassion as a trait. We did not find an effect of compassion on DNAmTL in the YFS data. Although DNAmTL outperforms leukocyte telomere length in predicting aging-related health conditions and mortality risk, the two are only moderately correlated (41). The fact that practicing compassion is obviously not the same thing as trait-like compassion might further explain the difference compared to previous studies.

There is a growing consensus that a *p* value close to 0.05 is too liberal for new discoveries to declare an association significant (63). At the same time, there is disagreement on the level of adjustment that is required when different tests of the same hypothesis are conducted or when highly correlated indicators are compared (64). When applying the most conservative Bonferroni correction for five comparisons, the null hypothesis of each DNA methylation-based epigenetic aging indicator would only be rejected if it has a value of *p*<0.01. The observed effect of compassion on DNAmPhenoAge should be interpreted as weak and would not survive testing for multiple correction. Further, not all of the analyses of the different aspects of the human aging process yield a significant result. The findings were also to some degree modified by variation in sample size due to non-response. Given that the single finding for one of the outcomes holds up depending on which control variables are regressed out and otherwise becomes marginally significant, the overall picture could also be interpreted as a null finding. The fact that changes in DNAm biomarkers are generally reversible (25) nonetheless indicates some practical significance of our findings.

In a smaller subsample the effect of compassion on DNAmPhenoAge was still visible when adjusting for epigenetic age assessed more than a decade earlier. This allows us to rule out to some degree the interpretation that epigenetic age predicts compassion, and makes the presence of spurious effects less likely.

### 4.1. Limitations and strengths

The unequal timing of the compassion measurements and the assessments of DNAm age in the study design of the YFS, and the difference in sample size, made it impractical to estimate whether the change in compassion over time has an effect on (change in) epigenetic aging. Another limitation is that compassion was measured only with a self-report methodology, even though with a validated and well-known personality instrument, the TCI (18, 43). The used compassion scale was not validated against other measures of compassion. However, there does not exist a golden standard for assessing dispositional compassion nor a measure exclusively developed for its assessment corresponding to our definition of compassion (14–16). Despite having a relatively stable disposition, compassion has an episodical emotional component (14), and people are not always committed enough to act compassionately (21). The current paper only measured compassion as a motivational personality trait, and thus the results for epigenetic aging might not be generalizable to the emotion of compassion and compassion-induced prosocial behavior.

Even though the analyses were based on a relatively large and representative sample, this does not permit to rule out the possibility of committing a Type II error, which is not finding a relationship that actually exist. More studies are needed to add more evidence on the potential link between prosocial behavior and the pace of epigenetic aging.

We presented data for compassion measurements spanning 4 years of aging and for a subsample with 25 years difference in DNAm age measurements. This allowed us to draw some causal inferences of the relationship between trait-like compassion and epigenetic aging and to shed new light on biological mechanisms that may lead to longevity. Five DNAm age indicators (DNAmAgeHorvath, IEAA_Hannum, EEAA_Hannum, DNAmPhenoAge, and DNAmTL) were used that had demonstrated high validity and predictive power in previous work (25, 26). We were further able to control for a wide range of potential confounders, including childhood and adulthood factors.

### 4.2. Conclusion

The motivational personality trait compassion for others predicted a less accelerated epigenetic age as assessed with DNAmPhenoAge when controlling for sex, socioeconomic status, BMI, and baseline epigenetic age. The current study is the first to demonstrate a biological aging pathway for how compassion for others might lead to longevity. It remains possible that a broader prosocial personality, in part, drives this association, as cooperativeness also predicted DNAmPhenoAge. The results of the role of compassion for others in epigenetic aging must however be interpreted with caution. The prediction was only marginally statistically significant in the sex-adjusted model. The second measurement of compassion for others was not related to a younger epigenetic age and none of the other four biomarkers of aging were predicted by compassion. The support of our data for an association of trait-like compassion with DNA methylation-based epigenetic aging was overall weak and thus requires replication. The presented findings are nonetheless interesting, and suggest that DNAmPhenoAge is more likely to be malleable by behavioral choices such as prosocial behavior than other tested DNA methylation-based indicators.

## Data availability statement

The Cardiovascular Risk In Young Finns study (YFS) dataset presented in this article is not readily available because it comprises health related participant data and their use is therefore restricted under the regulations on professional secrecy (Act on the Openness of Government Activities, 612/1999) and on sensitive personal data (Personal Data Act, 523/1999, implementing the EU data protection directive 95/46/EC). Due to these legal restrictions, the data from this study can not be stored in public repositories or otherwise made publicly available. Requests to access the datasets should be directed to the chairman of the publication committee (Prof Mika Kähönen, Tampere University, Finland).

## Ethics statement

The 30 year follow-up of the YFS was approved by the ethics committee of the University of Turku [vernacular institution name: Varsinais-Suomen sairaanhoitopiirin kuntayhtymä, Eettinen toimikunta, Meeting Number 9/2010; study name, “Lasten sepelvaltimotaudin riskitekijät projekti (Laseri) 30-vuotis seurantatutkimus, 25.8.2010”]. The YFS was conducted according to the guidelines of the Helsinki declaration. Written informed consent to participate in the YFS was obtained.

## Author contributions

HD, LK-J, SM, PM, AS, CC, IZ, MK, MHu, OR, TL, and MHi have made a substantial contribution to designing or carrying out the research, writing or revising the manuscript, or providing guidance on the execution of the research. The analyses have been conducted by HD, SM, and PM. All authors contributed to the article and approved the submitted version.

## Funding

This study was supported financially by the Academy of Finland (MHi, grant number 308676). The Young Finns Study has been financially supported by the Academy of Finland: grants 286284, 134309 (Eye), 126925, 121584, 124282, 129378 (Salve), 117787 (Gendi), 41071 (Skidi), and 322098; the Social Insurance Institution of Finland; Competitive State Research Financing of the Expert Responsibility area of Kuopio, Tampere and Turku University Hospitals (grant X51001); Juho Vainio Foundation; Paavo Nurmi Foundation; Finnish Foundation for Cardiovascular Research; Finnish Cultural Foundation; The Sigrid Juselius Foundation; Tampere Tuberculosis Foundation; Emil Aaltonen Foundation; Yrjö Jahnsson Foundation; Signe and Ane Gyllenberg Foundation (TL); Diabetes Research Foundation of the Finnish Diabetes Association; and EU Horizon 2020 (grant 755320 for TAXINOMISIS and grant 848146 for AITION); and European Research Council (grant 742927 for MULTIEPIGEN project); Tampere University Hospital Supporting Foundation, The Finnish Society of Clinical Chemistry (TL).

## Conflict of interest

PM and TL are employed by the company Fimlab Laboratories Oy.

The remaining authors declare that the research was conducted in the absence of any commercial or financial relationships that could be construed as a potential conflict of interest.

## Publisher’s note

All claims expressed in this article are solely those of the authors and do not necessarily represent those of their affiliated organizations, or those of the publisher, the editors and the reviewers. Any product that may be evaluated in this article, or claim that may be made by its manufacturer, is not guaranteed or endorsed by the publisher.

## Footnotes

^1^ http://dnamage.genetics.ucla.edu/home

## References

[1] Butler SM Snowdon DA. Trends in mortality in older women: findings from the nun study. J Gerontol B Psychol Sci Soc Sci. (1996) 51:S201–8. doi: 10.1093/geronb/51B.4.S201, PMID: 8673649

[2] Hilbrand S Coall DA Gerstorf D Hertwig R. Caregiving within and beyond the family is associated with lower mortality for the caregiver: a prospective study. Evol Hum Behav. (2017) 38:397–403. doi: 10.1016/j.evolhumbehav.2016.11.010

[3] O’Reilly D Connolly S Rosato M Patterson C. Is caring associated with an increased risk of mortality? A longitudinal study. Soc Sci Med. (2008) 67:1282–90. doi: 10.1016/j.socscimed.2008.06.025 18667262

[4] Poulin MJ Holman EA. Helping hands, healthy body? Oxytocin receptor gene and prosocial behavior interact to buffer the association between stress and physical health. Horm Behav. (2013) 63:510–7. doi: 10.1016/j.yhbeh.2013.01.004, PMID: 23354128

[5] Konrath S Fuhrel-Forbis A Lou A Brown S. Motives for volunteering are associated with mortality risk in older adults. Health Psychol. (2012) 31:87–96. doi: 10.1037/a0025226, PMID: 21842999

[6] Chapman BP Fiscella K Kawachi I Duberstein PR. Personality, socioeconomic status, and all-cause mortality in the United States. Am J Epidemiol. (2010) 171:83–92. doi: 10.1093/aje/kwp323, PMID: 19965888 PMC2800299

[7] Chapman BP Roberts B Duberstein P. Personality and longevity: knowns, unknowns, and implications for public health and personalized medicine. J Aging Res. (2011) 2011:759170. doi: 10.4061/2011/759170, PMID: 21766032 PMC3134197

[8] Friedman HS Kern ML. Personality, well-being, and health. Annu Rev Psychol. (2014) 65:719–42. doi: 10.1146/annurev-psych-010213-115123 24405364

[9] Weiss A Costa PT. Domain and facet personality predictors of all-cause mortality among medicare patients aged 65 to 100. Psychosom Med. (2005) 67:724–33. doi: 10.1097/01.psy.0000181272.58103.18, PMID: 16204430

[10] Dobewall H Saarinen A Lyytikäinen LP Keltikangas-Järvinen L Lehtimäki T Hintsanen M. Functional polymorphisms in oxytocin and dopamine pathway genes and the development of dispositional compassion over time: the Young Finns study. Front Psychol. (2021) 12:944. doi: 10.3389/fpsyg.2021.576346, PMID: 33897514 PMC8060576

[11] Kim JJ Cunnington R Kirby JN. The neurophysiological basis of compassion: an fMRI meta-analysis of compassion and its related neural processes. Neurosci Biobehav Rev. (2020) 108:112–23. doi: 10.1016/j.neubiorev.2019.10.023, PMID: 31697955

[12] Saarinen A Keltikangas-Järvinen L Dobewall H Ahola-Olli A Salmi M Lehtimäki T. Risky emotional family environment in childhood and depression-related cytokines in adulthood: the protective role of compassion. Dev Psychobiol. (2021b) 63:1190–201. doi: 10.1002/dev.22070, PMID: 33421111

[13] Warrier V Toro R Chakrabarti B iPSYCH-Broad autism group Børglum AD Grove J. Genome-wide analyses of self-reported empathy: correlations with autism, schizophrenia, and anorexia nervosa. Transl Psychiatry. (2018) 8:1–10. doi: 10.1038/s41398-017-0082-6, PMID: 29527006 PMC5845860

[14] Goetz JL Keltner D Simon-Thomas E. Compassion: an evolutionary analysis and empirical review. Psychol Bull. (2010) 136:351–74. doi: 10.1037/a0018807, PMID: 20438142 PMC2864937

[15] Lazarus RS. Emotion & Adaptation. New York, N.Y: In Oxford University Press (1991). Available at: https://psycnet.apa.org/record/1991-98760-000

[16] Gilbert P. The evolution and social dynamics of compassion. Soc Personal Psychol Compass. (2015) 9:239–54. doi: 10.1111/spc3.12176

[17] Garcia D Lester N Cloninger KM Cloninger CR. Cooperativeness In: Zeigler-Hill V Shackelford T, editors. Encyclopedia of personality and individual differences. Cham, Switzerland: Springer (2017). 1–3.

[18] Cloninger CR Svrakic DM Przybeck TR. A psychobiological model of temperament and character. Arch Gen Psychiatry. (1993) 50:975–90. doi: 10.1001/archpsyc.1993.01820240059008 8250684

[19] Eisenberg N Eggum ND Di Giunta L. Empathy-related responding: associations with prosocial behavior, aggression, and intergroup relations. Soc Issues Policy Rev. (2010) 4:143–80. doi: 10.1111/j.1751-2409.2010.01020.x, PMID: 21221410 PMC3017348

[20] Rudolph U Roesch S Greitemeyer T Weiner B. A meta-analytic review of help giving and aggression from an attributional perspective: contributions to a general theory of motivation. Cognit Emot. (2004) 18:815–48. doi: 10.1080/02699930341000248

[21] Poulin M.J. (2017). To help or not to help: goal commitment and the goodness of compassion. In Seppälä J.R. E.M Simon-Thomas E. Brown S.L. Worline M.C. Cameron L Doty (Eds.), The Oxford Handbook of Compassion Science (pp. 355–367). New York: Oxford University Press.

[22] Lee EE Govind T Ramsey M Wu TC Daly R Liu J. Compassion toward others and self-compassion predict mental and physical well-being. Transl Psychiatry. (2021) 11:1–9. doi: 10.1038/s41398-021-01491-8, PMID: 34282145 PMC8287292

[23] Saarinen AI Keltikangas-Järvinen L Pulkki-Råback L Cloninger CR Elovainio M Lehtimäki T. The relationship of dispositional compassion with well-being: a study with a 15-year prospective follow-up. J Posit Psychol. (2019) 15:806–20. doi: 10.1080/17439760.2019.1663251

[24] Martin P Da Rosa G Siegler IC Davey A MacDonald M Poon LW. Personality and longevity: findings from the Georgia centenarian study. Age. (2006) 28:343–52. doi: 10.1007/s11357-006-9022-8, PMID: 22253500 PMC3259159

[25] Horvath S Raj K. DNA methylation-based biomarkers and the epigenetic clock theory of ageing. Nat Rev Genet. (2018) 19:371–84. doi: 10.1038/s41576-018-0004-3, PMID: 29643443

[26] Jylhävä J Pedersen NL Hägg S. Biological age predictors. EBioMedicine. (2017) 21:29–36. doi: 10.1016/j.ebiom.2017.03.046, PMID: 28396265 PMC5514388

[27] Levine ME Lu AT Quach A Chen BH Assimes TL Bandinelli S. An epigenetic biomarker of aging for lifespan and healthspan. Aging. (2018) 10:573–91. doi: 10.18632/aging.101414, PMID: 29676998 PMC5940111

[28] Blackburn EH. Structure and function of telomeres. Nature. (1991) 350:569–73. doi: 10.1038/350569a0 1708110

[29] Blasco MA. Telomere length, stem cells and aging. Nat Chem Biol. (2007) 3:640–9. doi: 10.1038/nchembio.2007.38 17876321

[30] Hannum G Guinney J Zhao L Zhang L Hughes G Sadda SV. Genome-wide methylation profiles reveal quantitative views of human aging rates. Mol Cell. (2013) 49:359–67. doi: 10.1016/j.molcel.2012.10.016, PMID: 23177740 PMC3780611

[31] Horvath S. DNA methylation age of human tissues and cell types. Genome Biol. (2013) 14:R115. doi: 10.1186/gb-2013-14-10-r115, PMID: 24138928 PMC4015143

[32] Horvath S. (2021). Tutorial for the online age calculator: estimate DNA methylation age. Retrieved from Available at: https://dnamage.genetics.ucla.edu/sites/all/files/tutorials/TUTORIALonlineCalculator.pdf

[33] Chen BH Marioni RE Colicino E Peters MJ Ward-Caviness CK Tsai PC. DNA methylation-based measures of biological age: meta-analysis predicting time to death. Aging. (2016) 8:1844–65. doi: 10.18632/aging.101020, PMID: 27690265 PMC5076441

[34] Marioni RE Harris SE Shah S McRae AF von Zglinicki T Martin-Ruiz C. The epigenetic clock and telomere length are independently associated with chronological age and mortality. Int J Epidemiol. (2016) 45:424–32. doi: 10.1093/ije/dyw041, PMID: 27075770 PMC4864882

[35] Chaix R Alvarez-López MJ Fagny M Lemee L Regnault B Davidson RJ. Epigenetic clock analysis in long-term meditators. Psychoneuroendocrinology. (2017) 85:210–4. doi: 10.1016/j.psyneuen.2017.08.016, PMID: 28889075 PMC5863232

[36] Gibbs WW. Biomarkers and ageing: the clock-watcher. Nature. (2014) 508:168–70. doi: 10.1038/508168a, PMID: 24717494

[37] Pommier E Neff KD Tóth-Király I. The development and validation of the compassion scale. Assessment. (2020) 27:21–39. doi: 10.1177/1073191119874108 31516024

[38] Le Nguyen KD Lin J Algoe SB Brantley MM Kim SL Brantley J. Loving-kindness meditation slows biological aging in novices: evidence from a 12 week randomized controlled trial. Psychoneuroendocrinology. (2019) 108:20–7. doi: 10.1016/J.PSYNEUEN.2019.05.020, PMID: 31185369

[39] Jacobs TL Epel ES Lin J Blackburn EH Wolkowitz OM Bridwell DA. Intensive meditation training, immune cell telomerase activity, and psychological mediators. Psychoneuroendocrinology. (2011) 36:664–81. doi: 10.1016/j.psyneuen.2010.09.010, PMID: 21035949

[40] Hintsanen M Gluschkoff K Dobewall H Cloninger CR Keltner D Saarinen A. Parent-child-relationship quality predicts offspring dispositional compassion in adulthood: a prospective follow-up study over three decades. Dev Psychol. (2019) 55:216–25. doi: 10.1037/dev0000633, PMID: 30431291

[41] Lu AT Seeboth A Tsai PC Sun D Quach A Reiner AP. DNA methylation-based estimator of telomere length. Aging. (2019) 11:5895–923. doi: 10.18632/aging.102173, PMID: 31422385 PMC6738410

[42] Raitakari OT Juonala M Rönnemaa T Keltikangas-Järvinen L Räsänen L Pietikäinen M. Cohort profile: the cardiovascular risk in Young Finns study. Int J Epidemiol. (2008) 37:1220–6. doi: 10.1093/ije/dym225, PMID: 18263651

[43] Vitoratou S Ntzoufras I Theleritis C Smyrnis N Stefanis NC. Temperament and character dimensions assessed in general population, in individuals with psychoactive substance dependence and in young male conscripts. Eur Psychiatry. (2015) 30:474–9. doi: 10.1016/j.eurpsy.2015.01.007, PMID: 25687735

[44] Cloninger CR Svrakic DM Bayon C Przybeck TR. Measurement of psychopathology as variants of personality In: Cloninger CR, editor. Personality and psychopathology. Washington, DC: American Psychiatric Association (1999). 33–65.

[45] Kananen L Marttila S Nevalainen T Kummola L Junttila I Mononen N. The trajectory of the blood DNA methylome ageing rate is largely set before adulthood: evidence from two longitudinal studies. Age. (2016) 38:65. doi: 10.1007/s11357-016-9927-9, PMID: 27300324 PMC5005919

[46] Simpkin AJ Howe LD Tilling K Gaunt TR Lyttleton O McArdle W. The epigenetic clock and physical development during childhood and adolescence: longitudinal analysis from a UK birth cohort. Int J Epidemiol. (2017) 46:549–58. doi: 10.1093/ije/dyw307, PMID: 28089957 PMC5722033

[47] Marioni RE Shah S McRae AF Chen BH Colicino E Harris SE. DNA methylation age of blood predicts all-cause mortality in later life. Genome Biol. (2015a) 16:25. doi: 10.1186/s13059-015-0584-6, PMID: 25633388 PMC4350614

[48] Marioni RE Shah S McRae AF Ritchie SJ Muniz-Terrera G Harris SE. The epigenetic clock is correlated with physical and cognitive fitness in the Lothian birth cohort 1936. Int J Epidemiol. (2015b) 44:1388–96. doi: 10.1093/ije/dyu277, PMID: 25617346 PMC4588858

[49] Perna L Zhang Y Mons U Holleczek B Saum KU Brenner H. Epigenetic age acceleration predicts cancer, cardiovascular, and all-cause mortality in a German case cohort. Clin Epigenetics. (2016) 8:64. doi: 10.1186/s13148-016-0228-z, PMID: 27274774 PMC4891876

[50] Carroll JE Irwin MR Levine M Seeman TE Absher D Assimes T. Epigenetic aging and immune senescence in women with insomnia symptoms: findings from the women’s health initiative study. Biol Psychiatry. (2017) 81:136–44. doi: 10.1016/j.biopsych.2016.07.008, PMID: 27702440 PMC5536960

[51] Jylhävä J Hjelmborg J Soerensen M Munoz E Tan Q Kuja-Halkola R. Longitudinal changes in the genetic and environmental influences on the epigenetic clocks across old age: evidence from two twin cohorts. EBioMedicine. (2019) 40:710–6. doi: 10.1016/j.ebiom.2019.01.040, PMID: 30704927 PMC6413471

[52] Carlson LE Beattie TL Giese-Davis J Faris P Tamagawa R Fick LJ. Mindfulness-based cancer recovery and supportive-expressive therapy maintain telomere length relative to controls in distressed breast cancer survivors. Cancer. (2015) 121:476–84. doi: 10.1002/cncr.29063, PMID: 25367403

[53] Pulkki-Råback L Elovainio M Hakulinen C Lipsanen J Hintsanen M Jokela M. Cumulative effect of psychosocial factors in youth on ideal cardiovascular health in adulthood the cardiovascular risk in young Finns study. Circulation. (2015) 131:245–53. doi: 10.1161/CIRCULATIONAHA.113.007104, PMID: 25583139

[54] Locke AE Kahali B Berndt SI Justice AE Pers TH Day FR. Genetic studies of body mass index yield new insights for obesity biology. Nature. (2015) 518:197–206. doi: 10.1038/nature14177, PMID: 25673413 PMC4382211

[55] Abelson JL Erickson TM Mayer SE Crocker J Briggs H Lopez-Duran NL. Brief cognitive intervention can modulate neuroendocrine stress responses to the Trier social stress test: buffering effects of a compassionate goal orientation. Psychoneuroendocrinology. (2014) 44:60–70. doi: 10.1016/J.PSYNEUEN.2014.02.016, PMID: 24767620 PMC4120861

[56] Pace TWW Negi LT Adame DD Cole SP Sivilli TI Brown TD. Effect of compassion meditation on neuroendocrine, innate immune and behavioral responses to psychosocial stress. Psychoneuroendocrinology. (2009) 34:87–98. doi: 10.1016/J.PSYNEUEN.2008.08.011, PMID: 18835662 PMC2695992

[57] Lebowitz MS Dovidio JF. Implications of emotion regulation strategies for empathic concern, social attitudes, and helping behavior. Emotion. (2015) 15:187–94. doi: 10.1037/a0038820, PMID: 25706828

[58] Alda M Puebla-Guedea M Rodero B Demarzo M Montero-Marin J Roca M. Zen meditation, length of telomeres, and the role of experiential avoidance and compassion. Mindfulness. (2016) 7:651–9. doi: 10.1007/s12671-016-0500-5, PMID: 27217844 PMC4859856

[59] Cosley BJ McCoy SK Saslow LR Epel ES. Is compassion for others stress buffering? Consequences of compassion and social support for physiological reactivity to stress. J Exp Soc Psychol. (2010) 46:816–23. doi: 10.1016/j.jesp.2010.04.008

[60] Saarinen A Keltikangas-Järvinen L Viding E Dobewall H Kaseva K Lehtimäki T. Compassion protects against vital exhaustion and negative emotionality. Motiv Emot. (2021a) 45:506–17. doi: 10.1007/s11031-021-09878-2, PMID: 34720257 PMC8550749

[61] Quach A Levine ME Tanaka T Lu AT Chen BH Ferrucci L. Epigenetic clock analysis of diet, exercise, education, and lifestyle factors. Aging. (2017) 9:419–46. doi: 10.18632/aging.101168, PMID: 28198702 PMC5361673

[62] Raffington L Belsky DW Kothari M Malanchini M Tucker-Drob EM Harden KP. Socioeconomic disadvantage and the pace of biological aging in children. Pediatrics. (2021) 147:e2020024406. doi: 10.1542/peds.2020-024406, PMID: 34001641 PMC8785753

[63] Benjamin DJ Berger JO Johannesson M Nosek BA Wagenmakers EJ Berk R. Redefine statistical significance. Nature Human. Behaviour. (2018) 2:6–10. doi: 10.1038/s41562-017-0189-z 30980045

[64] Rubin M. Do p values lose their meaning in exploratory analyses? It depends how you define the familywise error rate. Rev Gen Psychol. (2017) 21:269–75. doi: 10.1037/gpr0000123

