# Supplementary material for: The relationship of trait-like compassion with epigenetic aging: The population-based prospective Young Finns Study
Source: Front Psychiatry. 2023 Apr 18;14:1018797. doi: 10.3389/fpsyt.2023.1018797 (PMC10151573; doi:10.3389/fpsyt.2023.1018797)
Supplement: Supplementary file 1 [file Table_1.DOCX]

Supplementary Material

Dobewall et al. “Compassion and epigenetic aging”

**Supplement Table S1.** Analysis of the Full Cooperativeness Scale in 1997 Predicting Five DNAm Epigenetic Aging Indicators in 2011, while Accounting for Sex (Model S1), Socioeconomic Status in Childhood and Adulthood, and Body-mass Index (Model S2).

|  | Model S1 |  |  |  | Model S1 |  |  |  |
| --- | --- | --- | --- | --- | --- | --- | --- | --- |
|  | Beta | p-value | R^2^ | n | Beta | p-value | R^2^ | n |
| *Epigenetic age acceleration (DNAmAge) Horvath 2011* | | | 2.5% | 1030 |  |  | 4.0% | 843 |
| Cooperativeness 1997 | -0.12 | .506 |  |  | -0.15 | .462 |  |  |
| *Intrinsic epigenetic age acceleration (IEAA) Hannum 2011* | | | 1.8% |  |  |  | 3.3% |  |
| Cooperativeness 1997 | -0.20 | .256 |  |  | -0.23 | .263 |  |  |
| *Extrinsic epigenetic age acceleration (EEAA) Hannum 2011* | | | 2.5% |  |  |  | 3.2% |  |
| Cooperativeness 1997 | -0.24 | .263 |  |  | -0.30 | .205 |  |  |
| *Phenotypic epigenetic age acceleration (DNAmPhenoAge) 2011* | | | 1.6% |  |  |  | 4.3% |  |
| Cooperativeness 1997 | -0.49 | .030 |  |  | -0.64 | .012 |  |  |
| *DNA methylation based indicator of telomere length (DNAmTL) 2011* | | | 2.3% |  |  |  | 2.6% |  |
| Cooperativeness 1997 | -0.00 | .791 |  |  | 0.01 | .501 |  |  |

***Note***. The Cooperativeness scale Cloninger’s biopsychological model of personality (Cloninger et al., 1993; Garcia et al., 2017) consists of 42 items and had an internal consistency of Cronbach’s alpha of 0.90 in 1997.

**Supplement Table S2.** Analysis of the Cooperativeness Subscale in 1997 Predicting Five DNAm Epigenetic Aging Indicators in 2011, while Accounting for Sex (Model S1), Socioeconomic Status in Childhood and Adulthood, and Body-mass Index (Model S2).

|  | Model S1 |  |  |  | Model S2 |  |  |  |
| --- | --- | --- | --- | --- | --- | --- | --- | --- |
|  | Beta | p-value | R^2^ | n | Beta | p-value | R^2^ | n |
| *Phenotypic epigenetic age acceleration (DNAmPhenoAge) 2011* | | | 1.5% | 1030 |  |  | 4.3% | 843 |
| Compassion (vs revengefulness) | -.34 | .050 |  |  | -.47 | .016 |  |  |
| *Phenotypic epigenetic age acceleration (DNAmPhenoAge) 2011* | | | 1.4% |  |  |  | 4.0% |  |
| Helpfulness (vs unhelpfulness) | -.26 | .130 |  |  | -.37 | .055 |  |  |
| *Phenotypic epigenetic age acceleration (DNAmPhenoAge) 2011* | | | 1.4% |  |  |  | 4.1% |  |
| Empathy (vs social disinterest) | -.28 | .119 |  |  | -.41 | .044 |  |  |
| *Phenotypic epigenetic age acceleration (DNAmPhenoAge) 2011* | | | 1.3% |  |  |  | 3.9% |  |
| Social acceptance (vs social intolerance) | -.18 | .286 |  |  | -.32 | .085 |  |  |
| *Phenotypic epigenetic age acceleration (DNAmPhenoAge) 2011* | | | 1.6% |  |  |  | 3.9% |  |
| Pure-hearted conscience (vs self-serving advantage) | -.38 | .030 |  |  | -.29 | .137 |  |  |

**Supplement Table S3.** Analysis Excluding the Revengefulness of the Compassion scale in 1997 Predicting Five DNAm Epigenetic Aging Indicators in 2011, while Accounting for Sex (Model S1), Socioeconomic Status in Childhood and Adulthood, and Body-mass Index (Model S2).

|  | Model S1 |  |  |  | Model S2 |  |  |  |
| --- | --- | --- | --- | --- | --- | --- | --- | --- |
|  | Beta | p-value | R^2^ | n | Beta | p-value | R^2^ | n |
| *Epigenetic age acceleration (DNAmAge) Horvath 2011* | | | 2.6% | 1030 |  |  | 4.0% | 843 |
| Positively worded items 1997 | -0.18 | .198 |  |  | -0.17 | .253 |  |  |
| *Intrinsic epigenetic age acceleration (IEAA) Hannum 2011* | | | 1.9% |  |  |  | 3.3% |  |
| Positively worded items 1997 | -0.17 | .192 |  |  | -0.19 | .204 |  |  |
| *Extrinsic epigenetic age acceleration (EEAA) Hannum 2011* | | | 2.4% |  |  |  | 3.0% |  |
| Positively worded items 1997 | 0.05 | .778 |  |  | 0.06 | .726 |  |  |
| *Phenotypic epigenetic age acceleration (DNAmPhenoAge) 2011* | | | 1.4% |  |  |  | 4.2% |  |
| Positively worded items 1997 | -0.29 | .092 |  |  | -0.44 | .023 |  |  |
| *DNA methylation based indicator of telomere length (DNAmTL) 2011* | | | 2.3% |  |  |  | 2.6% |  |
| Positively worded items 1997 | -0.00 | .776 |  |  | 0.00 | .518 |  |  |

***Note***. The five items capturing the compassion pole had an internal consistency of Cronbach’s alpha of 0.73 in 1997. Reversed revengefulness items excluded.

**Supplement Table S4.** Subsample Analysis of Compassion in 1997 Predicting Five DNAm Epigenetic Aging Indicators in 2011, while Accounting for Baseline Levels of the Outcome Measured 25 Years Earlier Additional to Sex (Model S3), Socioeconomic Status in Childhood and Adulthood, and Body-mass Index (Model S4).

|  | Model S3 |  |  |  | Model S4 |  |  |  |
| --- | --- | --- | --- | --- | --- | --- | --- | --- |
|  | Beta | p-value | R^2^ | n | Beta | p-value | R^2^ | n |
| *Epigenetic age acceleration (DNAmAge) Horvath 2011* | | | 48% | 81 |  |  | 47% | 66 |
| DNAmAgeHorvath 1986 | 0.66 | <.001 |  |  | 0.59 | <.001 |  |  |
| Compassion 1997 | 0.07 | .849 |  |  | 0.34 | .443 |  |  |
| *Intrinsic epigenetic age acceleration (IEAA) Hannum 2011* | | | 45% |  |  |  | 47% |  |
| IEAA_Hannum 1986 | 0.58 | <.001 |  |  | 0.53 | <.001 |  |  |
| Compassion 1997 | 0.20 | .609 |  |  | 0.42 | .337 |  |  |
| *Extrinsic epigenetic age acceleration (EEAA) Hannum 2011* | | | 29% |  |  |  | 26% |  |
| EEAA_Hannum 1986 | 0.52 | <.001 |  |  | 0.39 | .001 |  |  |
| Compassion 1997 | -0.31 | .559 |  |  | 0.14 | .810 |  |  |
| *Phenotypic epigenetic age acceleration (DNAmPhenoAge) 2011* | | | 31% |  |  |  | 35% |  |
| DNAmPhenoAge 1986 | 0.59 | <.001 |  |  | 0.61 | <.001 |  |  |
| Compassion 1997 | -1.50 | .007 |  |  | -1.18 | .052 |  |  |
| *DNA methylation based indicator of telomere length (DNAmTL) 2011* | | | 53% |  |  |  | 50% |  |
| DNAmTL 1986 | 0.76 | <.001 |  |  | 0.75 | <.001 |  |  |
| Compassion 1997 | 0.02 | .291 |  |  | 0.01 | .676 |  |  |

***Note*.** A smaller subsample of 137 participants donated blood samples in the year 1986 (age15-24 years). The gold standard correlation was r~97. In 93,4% blood PBMC instead of whole blood was predicted, and in 100% of the samples, sex was predicted correctly. As indicated in our previous study (Kananen et al., 2016), the included DNAm age measures were relatively stable from 1986 to 2011, r = .42 (DNAmPhenoAge) - .72 (p < .001) (DNAmTL), which is noteworthy given that these correlations represents 25 years the DNA methylome of an individual had aged.

**References Supplement**

Cloninger, C.R., Svrakic, D.M., & Przybeck, T.R. (1993). A psychobiological model of temperament and character. *Archives of General Psychiatry*, 50(12), 975–990. https://doi.org/10.1001/archpsyc.1993.01820240059008

Garcia, D., Lester, N., Cloninger, K.M., & Cloninger, C.R. (2017). Cooperativeness. In V. Zeigler-Hill & T. Shackelford (Eds.), *Encyclopedia of Personality and Individual Differences* (pp. 1–3). Cham, Switzerland: Springer.

Kananen, L., Marttila, S., Nevalainen, T., et al. (2016). The trajectory of the blood DNA methylome ageing rate is largely set before adulthood: evidence from two longitudinal studies. *Age*, 38(3), 65. https://doi.org/10.1007/s11357-016-9927-9
